# Results of a Targeted Glaucoma Screening Project Conducted Outside Formal Healthcare Settings in Croatia

**DOI:** 10.3390/jcm14238423

**Published:** 2025-11-27

**Authors:** Sonja Jandroković, Dina Lešin Gaćina, Sania Vidas Pauk, Martina Tomić, Tomislav Bulum, Bruno Rački, Vedrana Aljinović-Vučić

**Affiliations:** 1 Department of Ophthalmology, Zagreb University Hospital Center, Kišpatićeva 12, 10000 Zagreb, Croatia; 2 School of Medicine, University of Zagreb, Šalata 3, 10000 Zagreb, Croatia; 3 Department of Ophthalmology, Vuk Vrhovac University Clinic for Diabetes, Endocrinology and Metabolic Diseases, Merkur University Hospital, Dugi dol 4a, 10000 Zagreb, Croatia; 4 Department of Diabetes and Endocrinology, Vuk Vrhovac University Clinic for Diabetes, Endocrinology and Metabolic Diseases, Merkur University Hospital, Dugi dol 4a, 10000 Zagreb, Croatia; 5 Jadran Galenski Laboratorij d.d., Ivana Lučića 2a, 10000 Zagreb, Croatia; 6 Medical Affairs Department, Jadran Galenski Laboratorij d.d., Svilno 20, 51000 Rijeka, Croatia; 7 Department of Basic and Clinical Pharmacology and Toxicology, School of Medicine, University of Rijeka, Braće Branchetta 20, 51000 Rijeka, Croatia

**Keywords:** glaucoma targeted screening project, pharmacy, Croatia, intraocular pressure, dry eye symptoms

## Abstract

**Background**: Although glaucoma is a leading cause of irreversible blindness, no universally accepted screening guidelines for this condition have been developed. A nationwide glaucoma screening project was developed with the aim of raising awareness and assessing regional differences in intraocular pressure (IOP) and dry eye symptoms, as well as identifying potential risk factors among various Croatian regions. **Methods**: Over six months in 2023, 68 ophthalmologists conducted screening at 68 pharmacies across Croatia. IOP was measured using calibrated iCare IC200 tonometers, and dry eye symptoms were assessed using the Schein questionnaire. **Results**: A total of 7348 adults participated (median age: 63 years). IOP values and rates of newly detected elevated IOP varied by region. The highest maximum IOP was recorded in Continental Croatia (43.3 mmHg), followed by Zagreb. Newly discovered elevated IOP was most common in Slavonia (9.47%) and Continental Croatia (7.58%). Higher IOP was more frequently detected in younger participants and those with a family history of glaucoma. In coastal regions (Dalmatia, Istria, and Kvarner), elevated IOP showed a significant association with dry eye symptoms. **Conclusions**: This was the largest community-based glaucoma screening initiative in Croatia. The findings reveal notable regional differences in IOP and associated risk factors, underscoring the value of targeted community screening in raising awareness and identifying individuals at risk.

## 1. Introduction

Glaucoma is one of the leading causes of irreversible visual impairment worldwide, representing a major public health challenge with substantial individual and socioeconomic consequences [1]. Despite the availability of effective treatments, nearly half of all affected individuals remain undiagnosed [2]. This high proportion of undetected cases is primarily due to the asymptomatic course of the disease in its early stages, during which progressive optic nerve damage and irreversible visual field loss develop silently over time [3]. In 2020, an estimated 76 million people were living with glaucoma globally, and projections suggest that this number will increase to 112 million by 2040, driven by population aging, increased life expectancy, and lifestyle factors [1]. This projected rise underscores the growing importance of early detection, timely intervention, and preventive eye care, particularly in aging societies such as those across Europe [4]. From a public health perspective, glaucoma contributes significantly to disability-adjusted life years (DALYs) associated with visual loss, and the global economic burden of its management is expected to rise in parallel with demographic trends [5].

The prevalence and distribution of glaucoma types vary geographically. Data on the prevalence of glaucoma in the Republic of Croatia are limited. A previously published study from the southern Mediterranean Dalmatian region reported a prevalence of 2.9% for acute PACG [6], whereas research from the lowland Slavonia region in eastern Croatia found an overall prevalence of 5.6% for POAG, which increased to 14% among individuals with a positive family history [7]. However, both studies were based on hospital records, which limits the generalizability of their findings and highlights the lack of population-level glaucoma data in Croatia.

Risk factors for the development and progression of glaucoma vary across populations, and ongoing research continues to identify additional ocular, systemic, and lifestyle-related determinants. Established risk factors include advanced age, non-White ethnicity, family history of glaucoma, pseudoexfoliation, optic disc hemorrhage, thinner central corneal thickness (CCT), and myopia [4,8]. To date, elevated intraocular pressure (IOP) remains the principal modifiable risk factor for the development and progression of glaucoma [3,8]. Accordingly, timely measurement of IOP and identification of individuals at risk are crucial for effective prevention and early intervention.

Dry eye disease (DED) symptoms represent another relevant concern in ocular health, as they are highly prevalent among adults and may coexist with—or even mask—early manifestations of glaucoma and other ocular disorders [9,10]. Combined evaluation of IOP and ocular surface symptoms may therefore provide a more integrated and clinically informative assessment of ocular health.

Although population-wide glaucoma screening is not currently considered cost-effective for the general population, European and American professional societies recommend targeted screening among high-risk individuals, including older adults and first-degree relatives of affected patients [11,12,13]. However, public awareness of glaucoma remains insufficient, with surveys indicating that between 20% and 70% of adults are unfamiliar with the disease, and many at-risk individuals—particularly older adults—do not undergo regular ophthalmologic examinations [14,15,16]. Therefore, alongside targeted screening efforts, improving public awareness and health literacy remains a key strategy for the prevention of glaucoma. Community-based public health initiatives can play a pivotal role in bridging this gap. Pharmacies, in particular, are increasingly recognized as accessible healthcare settings for preventive screening, health promotion, and patient education [17,18]. Pharmacists often serve as the first point of contact for individuals experiencing ocular discomfort or seeking advice about eye health, and their collaboration with ophthalmologists can enhance disease awareness, facilitate early risk identification, and ensure timely referral for specialist evaluation.

The present nationwide project was established as a public health initiative in Croatia, integrating pharmacists and ophthalmologists within a collaborative model outside formal healthcare setting. Its primary aim was to enhance public awareness of glaucoma, promote early detection, and identify individuals at increased risk across the country. Secondary objectives included assessing regional variations in IOP values and self-reported dry eye symptoms and identifying associated risk factors relevant to the Croatian population.

## 2. Materials and Methods

### 2.1. Study Design and Participants

This project was conducted as a cross-sectional study in collaboration between ophthalmologists and pharmacists, at the initiative and with the support of Jadran Galenski Laboratorij d.d. (JGL). JGL helped to distribute the project information leaflets and lent tonometers for measuring intraocular pressure. However, the company JGL excluded itself from the analysis and interpretation of the results, in order to avoid any conflict of interest.

It was performed over a six-month period, from 1 April to 1 October 2023, across the Republic of Croatia. A total of 68 ophthalmologists collaborated with 68 pharmacies, strategically selected due to their accessibility to the general public. Although Croatia is administratively divided into 21 counties, for the purpose of this study the participating pharmacies were grouped according to the country’s traditional historical and cultural regions: Continental Croatia, Istria and Kvarner, Slavonia, and Dalmatia [19]. This regional classification allowed for the comparative analysis of the IOP distribution and symptom profiles across different geographic and demographic settings.

This study was conducted in accordance with the Declaration of Helsinki. It was approved by the Pablo Pharmacies’ Ethics Committee (protocol number 25-20-003/2023, approval date: 21 February 2023). All study participants received oral information about the project and signed a written informed consent form.

The project participants were all adults (over 18 years old) of both genders, who visited local pharmacies during the specified afternoon hours within the designated period, either after receiving information about the project or for some other purpose. Ophthalmologists and pharmacists provided detailed explanations regarding the project’s aims and methods to everyone. For those who chose to participate and signed the informed consent form, ophthalmologists collected demographic and medical history data, then asked about symptoms of dry eye and measured the intraocular pressure.

### 2.2. Demographic Data and Medical History

The demographic data and medical history of participants were assessed, including their age at the time of this study, gender, and personal and family history of glaucoma. All participants were asked if they had previously been diagnosed with glaucoma and used antiglaucoma medication regularly or had family members with glaucoma.

### 2.3. Measurement of the Intraocular Pressure

Intraocular pressure was measured using calibrated iCare IC200 tonometers (iCare Finland Oy)—rebound handheld devices that provide a practical and reliable means for IOP assessment in a community-based setting and offer clear advantages in terms of portability, ease of use, and patient comfort. Two measurements were taken and, if they differed by more than 2 mmHg, a third reading was obtained. The average value was used in the analysis. Previous studies have demonstrated good agreement between rebound tonometry and Goldmann applanation tonometry, with reported deviations typically within ± 2 mmHg under standard conditions [20,21]. For safety and practical reasons, the threshold for elevated IOP in this project was set at ≥20 mmHg, although the European Glaucoma Society (EGS) Guidelines define the upper limit of normal IOP as 21 mmHg [8]. This slightly lower cutoff was applied to minimize the risk of missed cases, considering the use of a handheld rebound tonometer, which may show minor variations compared with Goldmann applanation tonometry. In addition, large population-based studies show that a substantial proportion of glaucoma cases occur at IOP values ≤ 21 mmHg [22,23]. Moreover, our screening activities were conducted predominantly in the afternoon, when pharmacy attendance is highest. As IOP typically peaks in the early morning hours and declines throughout the day [24], afternoon measurements are likely to underestimate peak values. To enhance the sensitivity of community-based screening and reduce the likelihood of missing individuals whose IOP may have been higher earlier in the day, we applied a slightly more conservative IOP cut-off. It should also be noted that pachymetry was not performed, IOP measurements were not adjusted for CCT, and each participant’s IOP was obtained during a single pharmacy visit. In addition, no confirmatory glaucoma diagnostics were carried out. Accordingly, an elevated IOP in this context should not be interpreted as definitive evidence of ocular hypertension or glaucoma.

### 2.4. Schein Questionnaire

A standardized Schein questionnaire was used to determine the presence of dry eye symptoms [25]. This six-item questionnaire evaluated participant-reported dry eye symptoms, which participants scored on a scale from 0 to 4 (0 = none, 1 = rarely, 2 = sometimes, 3 = often, and 4 = all the time). Higher total scores indicated more problems or symptoms, ranging from 0 to 24. The questionnaire has been validated and translated into the Croatian language, and it is commonly used in the routine clinical work of Croatian ophthalmologists. The main advantages are that it is practical, concise, straightforward, and understandable to all participants, especially older people, and it is not protected by copyright, making it widely available without restriction. In this project, due to the large number of participants and centers included, the total score on the Schein questionnaire and the degree of dry eye disease could not be determined for each participant. Instead, participants who answered one or more questions positively in the Schein questionnaire were considered to have dry eye symptoms, while those who answered all questions negatively were considered to have no dry eye symptoms.

### 2.5. Statistical Analysis

Statistical analysis was performed and graphs were created using Statistica^TM^ 14.0.1.25 (TIBCO Software Inc., Palo Alto, CA, USA). The Kolmogorov–Smirnov test was used to test the data distribution. The descriptive results are shown as medians (range, min–max) for continuous variables and numbers (percentages) for categorical variables. Differences in continuous data distributions were evaluated using the Kruskal–Wallis ANOVA test, and post hoc analysis was performed with multiple comparisons of the Kruskal–Wallis test when necessary. The Chi-square test was used for categorical data. The Spearman Rank Order correlation test was used to assess the strength and direction of associations between the examined variables. In all analyses, the level of statistical significance was set at a *p*-value of 0.05.

## 3. Results

### 3.1. Demographic Data

A total of 7348 participants (2171 males and 5177 females) with a median age of 63 years (range: 19–91) took part in this glaucoma screening project. Table 1 presents the regional distribution of participants alongside Croatia’s mid-2021 population estimate [17]. Employees of JGL d.d.—Svilno were significantly younger (typically of working age, with a median age of 39.5 years), compared to 61 to 65 years for participants from other regions (Kruskal–Wallis ANOVA test, H = 258.093, *p* < 0.001; Multiple Comparisons, z > 11.828, *p* < 0.001). No significant age differences were observed among other regions (*p* > 0.05), nor did the ratio of women to men differ significantly across regions (*p* > 0.05).

Summary: 7348 participants (2171 males and 5177 females) with a median age of 63 years took part in this glaucoma screening project.

### 3.2. Medical History of Glaucoma and Dry Eye Symptoms

Among all project participants, 536 (7.28%) had a prior glaucoma diagnosis and used antiglaucoma medications, while 718 (9.76%) had a positive family history of glaucoma. The regional distributions of these findings are presented in Table 2. The highest prior glaucoma diagnosis rate was in Istria and Kvarner (12.65%), followed by Dalmatia (9.59%), and the lowest among JGL employees (3.82%) (*p* < 0.001). A positive family history of glaucoma was most common among JGL employees (20.61%), followed by Istria and Kvarner (12.57%), and rarest in Continental Croatia (6.93%) (*p* < 0.001).

Dry eye disease, assessed using the Schein questionnaire for dry eye symptoms, was present in 2530 participants (Schein questionnaire score ≥ 1, 34.43%), while 4818 participants (65.57%) were symptom-free (Schein questionnaire score 0). The highest prevalence of dry eye symptoms was in Dalmatia (46.56%), followed by Istria and Kvarner (42.72%), with rates below 40% in other regions and the lowest among JGL employees (24.30%; *p* < 0.001); see Table 2.

Summary: Among all project participants, 536 (7.28%) had a prior glaucoma diagnosis and used antiglaucoma medications, while 718 (9.76%) had a positive family history of glaucoma. Dry eye symptoms were present in 2530 (34.43%) participants.

### 3.3. Intraocular Pressure and Prevalence of Newly Discovered Elevated Intraocular Pressure

**Figure 1:**
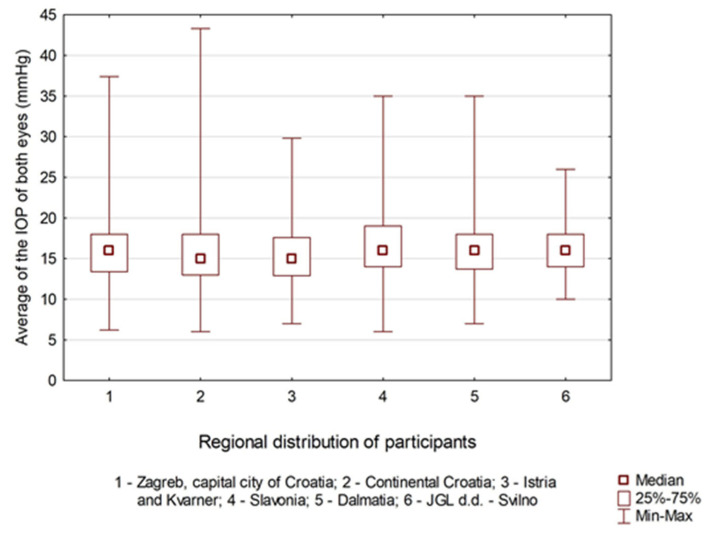
Average of the intraocular pressure (IOP) of both eyes across regions.

In this project involving 14,696 eyes, the median IOP was 15.8 mmHg (range: 6.0–43.3 mmHg). The maximum recorded IOP values showed regional variation: Continental Croatia reported the highest maximum IOP (43.3 mmHg), followed by Zagreb (37.4 mmHg). Both Istria and Kvarner, as well as Dalmatia, had a maximum of 35.0 mmHg, while JGL employees recorded the lowest maximum IOP of 26.0 mmHg (Kruskal–Wallis ANOVA test, *p* < 0.001; Multiple Comparisons, *p* < 0.033); see Figure 1.

**Figure 2:**
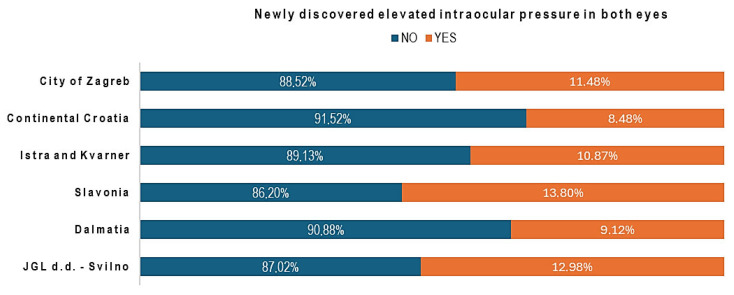
The percentage of newly discovered elevated intraocular pressure in both eyes.

Newly discovered elevated IOP was found in 495 participants (6.74%) in one eye and in 769 participants (10.47%) in both eyes. For one eye, it was most prevalent in Slavonia (9.47%), followed by Continental Croatia (7.58%), and least common among JGL employees (5.34%) (Chi-square test, χ^2^ = 12.153, *p* < 0.001). In other regions, it was less than 6.50%, without a significant difference between regions (*p* > 0.05). The regional distribution of newly discovered elevated IOP in both eyes showed variation, with the highest rate also in Slavonia (13.80%). JGL employees followed at 12.98%, then Zagreb (11.48%), and Istria and Kvarner (10.87%). In Dalmatia and Continental Croatia, the percentage was below 10.00% (Chi-square test, χ^2^ = 20.533, *p* < 0.001); see Figure 2.

Summary: In this project involving 14,696 eyes, the median IOP was 15.8 mmHg. Newly discovered elevated IOP in one eye was found in 495 participants (6.74%) and in both eyes in 769 participants (10.47%).

### 3.4. Correlations of Newly Discovered Elevated Intraocular Pressure and IOP Value in Both Eyes with Demographic and Clinical Characteristics

In all project participants, newly discovered elevated IOP was mainly found in younger individuals (R = −0.046859, *p* < 0.001) and in those with a positive family history of glaucoma (R = 0.063681, *p* < 0.001). The highest IOP value in both eyes was also noted in younger individuals (R = −0.054827, *p* < 0.001), particularly women (R = 0.046028, *p* < 0.001) with those with a positive family history of glaucoma (R = 0.072321, *p* < 0.001), as determined by the Spearman Rank Order correlation test.

When the correlation analysis was conducted by region, the following results emerged. In Zagreb, newly discovered elevated IOP in both eyes was most commonly found in women and those with dry eye disease. In Continental Croatia, it was noted in participants with a positive family history of glaucoma regardless of gender while, in Slavonia, higher rates of newly discovered elevated IOP were observed in women with a positive family history of glaucoma. Continuing from these findings, the highest IOP in both eyes was most frequently noted in participants with a positive family history of glaucoma in Zagreb, Continental Croatia, and Slavonia. In Zagreb, in addition to family history, the highest IOP was more often observed in women. In Continental Croatia, younger participants demonstrated higher IOP while, in Slavonia, both younger age and female gender were associated with higher IOP values (Table 3).

In the Adriatic regions, the results were somewhat different. In both Dalmatia and the Istria and Kvarner regions, we observed the expected positive association between a family history of glaucoma and IOP values, as well as newly discovered elevated IOP in both eyes. However, the newly discovered cases of elevated IOP in these regions showed a significant positive correlation with dry eye disease (Dalmatia R = 0.051454, *p* = 0.033; Istria and Kvarner R = 0.051621, *p* = 0.029) (Table 4). In Istria and Kvarner, higher IOP and more frequent newly discovered elevated IOP in both eyes were also observed among younger participants. In JGL employees in Svilno, the only significant association found was higher IOP values in both eyes among participants without a family history of glaucoma. Other correlations were not significant (Table 4).

Summary: In all project participants, newly discovered elevated IOP and the highest IOP values were primarily observed in younger individuals and in those with a positive family history of glaucoma. However, in Adriatic regions, newly identified cases of elevated IOP also showed a significant positive correlation with dry eye disease.

## 4. Discussion

This nationwide targeted glaucoma screening initiative, implemented through collaboration between ophthalmologists and pharmacists, represents the largest community-based ophthalmologic screening effort conducted in Croatia to date. This study aimed to increase public awareness of glaucoma and identify potential risk factors associated with elevated IOP within the Croatian population. To achieve these goals, IOP measurements and assessments of ocular dryness symptoms were performed among adults attending community pharmacies across the country.

This project provides valuable insight into the regional distribution of IOP, newly discovered elevated IOP, and associated risk factors. Slavonia—a continental lowland region—demonstrated the highest maximum IOP values (up to 43.3 mmHg) and the highest rates of newly discovered elevated IOP in both eyes (13.8%). This regional pattern may reflect the impact of both demographic and environmental influences, including higher prevalence of systemic hypertension, different dietary patterns, and variations in health-related behaviors [26]. These findings correspond with earlier evidence of regional differences in glaucoma risk factors in Croatia, with hospital-based data revealing increased rates of POAG in the eastern lowlands [7]. In contrast, coastal regions such as Dalmatia and Istria and Kvarner showed lower maximum IOP values but a higher prevalence of dry eye symptoms. The overall prevalence of newly discovered elevated IOP in both eyes observed in this project, at around 10%, aligns with findings from population-based studies [27,28,29]. Elevated IOP remains the primary risk factor for the development and progression of POAG, and the principal objective of OH management is to prevent its conversion to manifest glaucoma. The finding that approximately 10% of this study’s participants had borderline or elevated IOP highlights a substantial reservoir of at-risk individuals who could benefit from further diagnostic evaluation and monitoring.

Considering demographic and clinical correlations, our analysis revealed several consistent trends across regions. Younger participants and those with a positive family history of glaucoma were more likely to present with newly discovered elevated IOP and higher IOP values. Family history has consistently been associated with a two- to four-fold increase in the risk of developing POAG, representing a strong and independent risk factor for the disease [30,31]. The negative correlation between age and IOP observed in this study contrasts with findings from several longitudinal studies reporting an age-related increase in IOP. This discrepancy may be explained by the early onset of elevated IOP in genetically predisposed individuals, who tend to develop ocular hypertension at a younger age. It may also reflect the greater motivation of younger, health-conscious individuals with a family history of glaucoma to participate in community screening initiatives. In addition, the possibility of selection bias cannot be excluded. Older individuals already diagnosed with glaucoma are more likely to be under regular ophthalmic follow-up and treated with IOP-lowering medications, resulting in pharmacologically controlled measurements and reducing their likelihood of participating in a pharmacy-based screening initiative. Conversely, younger and previously undiagnosed individuals may present with higher untreated IOP values, thereby contributing to the observed inverse correlation between age and IOP. Moreover, the predominance of female participants, as well as higher IOP values observed among women in Zagreb, Continental Croatia, and Slavonia, could also partly result from gender differences in healthcare-seeking behavior, with women being more likely to participate in preventive health activities. In addition, hormonal factors and postmenopausal changes may contribute to variations in IOP and ocular surface health [32,33,34]. Overall, 34.4% of participants reported symptoms consistent with dry eye symptoms—a prevalence that falls within the global range of 5–50% reported in adults [35].

Among regions, the highest rates of self-reported dry eye symptoms were observed in Dalmatia, followed by Istria and Kvarner. The observed association between OH and dry eye symptoms in these Adriatic regions likely reflects the combined influence of environmental and climatic factors on ocular surface health and IOP regulation. The TFOS Lifestyle Report highlights that environmental conditions exert a substantial and well-established influence on the ocular surface [36]. Climate-related factors such as temperature, relative humidity, wind exposure, altitude, and ultraviolet (UV) radiation can destabilize the tear film, accelerate evaporation, and promote inflammatory responses that predispose individuals to ocular surface dysfunction. Seasonal and geographical variability further modulates the severity of symptoms, with dry, windy, or high-insolation environments exacerbating tear film instability and increasing the burden of dry eye disease. These environmental drivers interact with demographic and lifestyle factors, underscoring the complex and multifactorial nature of ocular surface responses described in the TFOS consensus. Within this framework, the warmer temperatures, lower humidity, elevated salinity, higher UV exposure, and stronger winds characteristic of Mediterranean coastal climates are known to intensify ocular surface stress and promote evaporative tear loss [37,38,39]. Increased tear evaporation may, in turn, influence corneal biomechanics and affect rebound or applanation tonometric measurements [40], potentially complicating the interpretation of IOP values and the assessment of glaucoma risk.

Furthermore, previous studies have reported seasonal variations in IOP, with higher values typically recorded in winter and lower values in summer, particularly among individuals with DED. As this study was conducted between April and October, during the spring and summer months, such environmental conditions may have contributed to enhanced evaporative stress and, consequently, to the observed association between OH and dry eye symptoms in coastal Croatia. Although causality cannot be established, these findings support the possibility that climate-related factors may indirectly influence the occurrence or detection of OH through their effects on the ocular surface.

Existing evidence suggests that universal, population-wide glaucoma screening provides limited clinical benefit and is not considered cost-effective. Similarly, the U.S. Preventive Services Task Force (USPSTF) concludes that evidence remains insufficient to recommend routine population-based screening in asymptomatic adults [12]. In contrast, our results highlight the public health value of a targeted, high-risk screening model conducted in accessible community settings such as pharmacies. This approach aligns with economic evaluations showing that targeted screening of high-risk individuals is more cost-effective than universal screening [41]. Moreover, the participants with elevated IOP in our study were provided with clear verbal recommendations for comprehensive ophthalmologic evaluation, including Goldmann applanation tonometry, optic nerve assessment, and visual field testing. However, the effectiveness of any community-based screening program ultimately depends on adherence to recommended follow-up. Referral adherence in this context is known to be suboptimal, with previous studies reporting that 30–70% of individuals identified as at risk do not attend subsequent examinations [42]. Although follow-up adherence was not assessed here, this limitation underscores the need for structured referral pathways, reminder systems, and closer coordination with primary eye-care providers to maximize the impact of such initiatives.

Our findings emphasize the public health value of a targeted screening approach for high-risk individuals that promotes preventive eye care and raises public awareness of glaucoma in non-clinical settings such as pharmacies. Pharmacies represent accessible, convenient, and familiar community points of first contact within the healthcare system. Consistent with previous community-based studies demonstrating the feasibility and impact of glaucoma awareness and screening initiatives outside traditional ophthalmology clinics [43,44,45], this project further extends that evidence. To the best of our knowledge, no comparable nationwide community-based glaucoma screening initiative involving direct collaboration between ophthalmologists and pharmacists has been previously reported. This makes the present study a unique and valuable contribution to public health efforts aimed at improving glaucoma awareness and facilitating early detection beyond conventional clinical environments.

Several limitations and methodological considerations should be acknowledged. The cross-sectional design of the study prevents causal inference. This project did not target a specific public awareness campaign; while it was prepared and organized by JGL, aimed at raising public awareness, it also relied on spontaneous visits to pharmacies. As most participants were self-selected volunteers visiting pharmacies, this could affect the study’s generalizability, and the results may not reflect the characteristics of the general Croatian population. Additionally, seasonal variation may have influenced the results. The use of the iCare IC200 rebound tonometer provided a practical and reliable approach to IOP measurement in a community setting. This method has shown good agreement with Goldmann applanation tonometry—the clinical gold standard—and offers advantages such as portability, ease of use, and patient comfort. However, minor deviations from Goldmann readings cannot be excluded, particularly in individuals with altered corneal biomechanics or extreme IOP values. Moreover, IOP values were not adjusted for central corneal thickness or diurnal fluctuation, and confirmatory glaucoma diagnostics were not performed, meaning that elevated IOP does not necessarily indicate ocular hypertension or glaucoma. Additionally, the Schein questionnaire—a validated and widely used instrument for the self-assessment of dry eye symptoms—was applied in a simplified format to identify participants with any indication of dry eye. Furthermore, the binary classification of dry eye (“any positive response = symptomatic”) simplified the analysis but may have led to a loss of information about severity, potentially overestimating prevalence or reducing the specificity of associations with IOP. Finally, some reported correlation coefficients were statistically significant but very weak, which may indicate the limited clinical relevance, even in large samples. While this approach facilitated the inclusion of a large number of participants, it limited the ability to assess disease severity or classify specific subtypes. Despite these limitations, the large sample size, standardized methodology, and nationwide scope of the project strengthen the reliability and generalizability of the findings.

## 5. Conclusions

In conclusion, this study demonstrated regional differences, with higher IOP and a greater proportion of newly discovered elevated IOP in Continental Croatia and Slavonia, whereas a positive correlation between newly discovered elevated IOP cases and dry eye symptoms was observed in coastal regions, suggesting potential demographic and environmental influences. This project provides valuable epidemiological insight into the nationwide distribution of glaucoma risk factors and offers guidance for future healthcare planning.

The findings highlight that pharmacy-based targeted screenings represent a promising approach for enhancing public awareness about glaucoma and identifying individuals at risk in a cost-effective and accessible manner. Expanding such initiatives may help to bridge the gap between clinical recommendations and the real-world need for earlier glaucoma diagnosis. Future research should include longitudinal follow-up across different seasons, incorporation of confirmatory imaging and functional assessments, and evaluation of the cost-effectiveness of community- and technology-based screening models within the Croatian healthcare system.

## Figures and Tables

**Table 1:** Number of participants included in the glaucoma screening project, along with Croatia’s mid-2021 population estimate, as per the Croatian Bureau of Statistics.

	Number of Participants	Mid-2021 Population Estimate
Republic of Croatia	7348	3,878,981
Zagreb, capital city of Croatia	2478	767,131
Continental Croatia	1500	874,903
Istria and Kvarner	893	460,656
Slavonia	626	665,858
Dalmatia	1720	795,118
JGL	131	824 (2024 est.)

Legend: JGL; Jadran Galenski Laboratorij d.d., Values are numbers.

**Table 2:** Regional distribution of participants based on prior glaucoma diagnosis, family history of glaucoma, and dry eye disease assessed by the Schein questionnaire for evaluation of dry eye symptoms.

	Prior Glaucoma Diagnosis (No/Yes)	Family History of Glaucoma (No/Yes)	Dry Eye Disease (No/Yes)
Zagreb, capital city	94.15/5.85	90.55/9.45	61.82/38.18
Continental Croatia	95.33/4.67	93.07/6.93	65.80/34.20
Istria and Kvarner	87.35/12.65	87.43/12.57	57.28/42.72
Slavonia	94.08/5.92	89.12/10.88	63.61/36.39
Dalmatia	90.41/9.59	90.06/9.94	53.44/46.56
JGL	96.18/3.82	79.39/20.61	75.70/24.30
χ^2^	78.491	40.841	122.644
*p*	<0.001	<0.001	<0.001

Legend: JGL; Jadran Galenski Laboratorij d.d., Values are percentages. χ^2^ indicates the Chi-square test (df = 5).

**Table 3:** Correlations of newly discovered elevated IOP and IOP value in both eyes with demographic and clinical characteristics of participants in Zagreb, Continental Croatia, and Slavonia.

	Age	Gender (m/f)	Family History of Glaucoma	Dry Eye Disease
Zagreb, capital city				
Newly discovered elevated IOP in both eyes	−0.036	0.057 *	0.033	0.041 *
IOP value in both eyes	−0.037	0.069 **	0.058 *	0.022
Continental Croatia				
Newly discovered elevated IOP in both eyes	−0.051	0.005	0.061 *	0.013
IOP value in both eyes	−0.083 *	0.049	0.063 *	0.046
Slavonia				
Newly discovered elevated IOP in both eyes	−0.066	0.087 *	0.086 *	−0.026
IOP value in both eyes	−0.111 *	0.093 *	0.079 *	−0.034

Legend: Values are Spearman’s R (rank) correlation coefficients. *, *p* < 0.05 and **, *p* < 0.001 (Spearman Rank Order correlation test); IOP, intraocular pressure.

**Table 4:** Correlations of newly discovered elevated IOP and IOP value in both eyes with demographic and clinical characteristics of participants in Dalmatia, Istria and Kvarner, and JGL.

	Age	Gender (m/f)	Family History of Glaucoma	Dry Eye Disease
Dalmatia				
Newly discovered elevated IOP in both eyes	−0.009	−0.028	0.086 **	0.051 *
IOP value in both eyes	−0.017	0.019	0.087 **	0.033
Istria and Kvarner				
Newly discovered elevated IOP in both eyes	−0.086 *	−0.007	0.108 *	0.052 *
IOP value in both eyes	−0.070 *	0.039	0.126 **	0.030
JGL				
Newly discovered elevated IOP in both eyes	0.056	−0.068	−0.084	0.049
IOP value in both eyes	0.038	−0.134	−0.173 *	−0.129

Legend: JGL, Jadran Galenski Laboratorij d.d., Values are Spearman’s R (rank) correlation coefficients. *, *p* < 0.05 and **, *p* < 0.001 (Spearman Rank Order correlation test); IOP, intraocular pressure.

## Data Availability

The data presented in this study are available at a specific request from the corresponding author.
